# *In vivo* analysis of insulin-like growth factor type 1 receptor humanized monoclonal antibody MK-0646 and small molecule kinase inhibitor OSI-906 in colorectal cancer

**DOI:** 10.3892/or.2013.2819

**Published:** 2013-10-25

**Authors:** PREMILA D. LEIPHRAKPAM, EKTA AGARWAL, MICHELLE MATHIESEN, KATIE L. HAFERBIER, MICHAEL G. BRATTAIN, SANJIB CHOWDHURY

**Affiliations:** Eppley Cancer Center, University of Nebraska Medical Center, Omaha, NE 68198-5950, USA

**Keywords:** colorectal cancer, IGF-1R, MK-0646, OSI-906, antitumor activity, X-linked inhibitor of apoptosis, cell survival

## Abstract

The development and characterization of effective anticancer drugs against colorectal cancer (CRC) is of urgent need since it is the second most common cause of cancer death. The study was designed to evaluate the effects of two IGF-1R antagonists, MK-0646, a recombinant fully humanized monoclonal antibody and OSI-906, a small molecule tyrosine kinase inhibitor on CRC cells. Xenograft study was performed on IGF-1R-dependent CRC cell lines for analyzing the antitumor activity of MK-0646 and OSI-906. Tumor proliferation and apoptosis were assessed using Ki67 and TUNEL assays, respectively. We also performed *in vitro* characterization of MK-0646 and OSI-906 treatment on CRC cells to identify mechanisms associated with drug-induced cell death. Exposure of the GEO and CBS tumor xenografts to MK-0646 or OSI-906 led to a decrease in tumor growth. TUNEL analysis showed an increase of approximately 45–55% in apoptotic cells in both MK-0646 and OSI-906 treated tumor samples. We report the novel finding that treatment with IGF-1R antagonists led to downregulation of X-linked inhibitor of apoptosis (XIAP) protein involved in cell survival and inhibition of cell death. In conclusion, IGF-1R antagonists (MK-0646 and OSI-906) demonstrated single agent inhibition of subcutaneous CRC xenograft growth. This was coupled to pro-apoptotic effects resulting in downregulation of XIAP and inhibition of cell survival. We report a novel mechanism by which MK-0646 and OSI-906 elicits cell death *in vivo* and *in vitro*. Moreover, these results indicate that MK-0646 and OSI-906 may be potential anticancer candidates for the treatment of patients with IGF-1R-dependent CRC.

## Introduction

The insulin-like growth factor receptor (IGF-1R) is a receptor tyrosine kinase that is widely expressed in normal human tissues and upregulated in a number of human cancers including colorectal cancer (CRC) (1–3). IGF-1R is comprised of three components, two extracellular α-chains, that are involved in ligand binding, two transmembrane spanning β-chains and an intracellular tyrosine kinase (2,4,5). Both IGF1 and IGF2 are ligands for IGF-1R and their binding induces receptor autophosphorylation at the tyrosine kinase domain, resulting in its activation by a conformational change leading to stimulation of signaling cascades, including PI3K/Akt and MAPK pathways (4–8). Activation of IGF-1R has been reported to stimulate oncogenic cellular processes, including aberrant cell survival mechanisms, transformation, motility, angiogenesis and metastasis (2,3,9). Previous work from our laboratory as well as other groups has shown that inhibition of IGF-1R has been shown to impede tumorigenesis in several human xenograft models (2,3,9,10).

IGF-IR plays a multifunctional role in human CRC growth and is widely regarded as an attractive target for anticancer drug treatment based on the observation that inhibition of IGF-1R function results in apoptosis and inhibition of tumor growth. Several pharmacological strategies are currently being adopted in clinical trials to disrupt the IGF-IF signaling pathway. This includes anti-receptor antibodies to reduce receptor expression, small-molecule IGF-1R kinase inhibitors, and targeting downstream IGF-1R signaling pathways with agents, such as Akt or mTOR inhibitors (9). In this study, we characterized the *in vivo* and *in vitro* effects of MK-0646, a novel IGF-1R recombinant humanized monoclonal antibody. It has been reported that MK-0646 binds to IGF-1R and triggers receptor internalization and degradation thereby blocking IGF-1 and II mediated cellular proliferation and survival (11). MK-0646 specifically targets IGF-1R and does not cross-react with the insulin receptor (12). It is in phase II clinical trial at present (13–16).

OSI-906 is a potent and highly selective small molecule tyrosine kinase inhibitor which binds dually to IGF-1R and IR and inhibits autophosphorylation (6,7). It is also in phase II clinical trials at present (16). Initiation of apoptosis and inhibition of cell proliferation following OSI-906 treatment appears to be directly linked to Akt inhibition in various tumor cell lines including lung, pancreatic and CRC cell lines (6,17). In addition, OSI-906 has shown potent antitumor activity *in vivo* in several xenograft models (18). Buck *et al* has shown that OSI-906 reduces tumorigenicity in GEO CRC xenografts (18). However, the signaling mechanisms associated with OSI-906-mediated cell death are poorly understood.

The goal of the present study was to compare the antagonistic effects of MK-0646 and OSI-906 *in vivo* and *in vitro* and characterize mechanisms associated with drug-induced cell death. We report for the first time the antitumor activity of MK-0646 in IGF-1R-dependent CRC cells and demonstrate that inhibition of IGF-1R leads to control of aberrant cell survival signaling through the downregulation of XIAP and induction of cell death.

## Materials and methods

### Cell lines

GEO and CBS cell lines used in this study were originally developed from primary CRC tumors and have been extensively characterized (19). Cells were maintained at 37°C in humidified atmosphere of 5% CO_2_ in a chemically defined serum-free medium consisting of McCoy's 5A medium (Sigma-Aldrich, St. Louis, MO, USA) supplemented with amino acids, pyruvate, vitamins, antibiotics and growth factors transferring (4 μg/ml; Sigma-Aldrich), insulin (20 μg/ml; Sigma-Aldrich), and EGF (10 ng/ml; R&D Systems) as previously described (20). Supplemented McCoy's medium (‘SM’) is McCoy's 5A medium supplemented with antibiotics and nutrients but lacking any growth factors. Cells were routinely subcultured with a 0.25% trypsin (Invitrogen, Carlsbad, CA, USA) in Joklik's medium (Invitrogen) containing 0.1% EDTA. When cells were under growth factor deprivation status (GFDS), they were cultured in SM medium without growth factor or serum supplements for the indicated time periods without medium change in between.

### Antibodies

IGF-1Rβ, pIGF-1Rβ (Y^1135^) and p21 antibodies were obtained from Cell Signaling Technology Inc. (Beverly, MA, USA). XIAP antibody was obtained from abcam. β-actin and GAPDH antibodies were from Sigma-Aldrich (St. Louis, MO, USA).

### Pharmacological antagonists

MK-0646 was provided by Merck & Co. (Whitehouse Station, NJ, USA) and OSI-906 was purchased from Chemitek, Indianapolis, IN, USA.

### Xenograft experiments

All experiments involving animals were approved by the University of Nebraska Medical Center Institutional Animal Care and Use Committee. The GEO and CBS cells were transfected with green fluorescence protein (GFP). Exponentially growing GFP-labeled GEO and CBS cells (~7 million cells/ml SF media) were inoculated subcutaneously onto the dorsal surfaces of athymic nude male mice and the growth of the tumor was monitored by biweekly measurements using a caliper. Once xenografts were established (~50–100 mm^3^), MK-0646 or OSI-906 treatment was initiated and continued for two weeks. MK-0646 was given by intraperitoneal (IP) injection weekly (20 mg/kg) on both GEO and CBS xenografted mice for three doses and formulation buffer was the vehicle. OSI-906 was given by daily oral gavage (40 mg/kg) on GEO xenografted mice and tartaric acid was the vehicle. Xenografts were harvested after 14 days of treatment for assessment of molecular effects by the two agents.

### Xenograft lysate preparation

Xenografts were harvested and snap frozen in liquid nitrogen and stored at −80°C. Xenografts were first washed in cold 5% PBS and collected in lysis buffer [50 mmol/l Tris (pH 7.4), 100 mmol/l NaCl, 1% NP40, 2 mmol/l EDTA, 0.1% SDS, 50 mmol/l NaF, 10 mmol/l Na_3_VO_4_, 1 mmol/l phenylmethylsulfonyl fluoride, 25 μg/ml β-glycerophosphate, and one protease inhibitor cocktail tablet from Roche]. Crude xenograft lysates were homogenized to shear DNA and lysed for 30 min on ice. Xenograft lysates were then cleared by centrifugation at 13000 rpm for 20 min at 4°C. Protein concentrations were determined by the Pierce bicinchonimic acid protein assay (Pierce Biotechnology, Inc., Rockford, IL, USA).

### Cell lysate preparation

GEO CRC cells were allowed to grow until 70–80% confluent in 100-mm culture plates and were treated with different concentrations of either MK-0646 or OSI-906 under growth factor deprivation status (GFDS) for 48 h. Cells were washed in cold 5% PBS and collected in lysis buffer. Crude cell lysates were homogenized using a 21-gauge needle to shear DNA and lysed for 30 min on ice. Cell lysates were then cleared by centrifugation at 13000 rpm for 20 min at 4°C. Protein concentrations were determined by the Pierce bicinchonimic acid protein assay (Pierce Biotechnology, Inc.).

### Western blot analysis

Protein (30–100 μg) was fractionated on an acrylamide denaturing gel and transferred onto a nitrocellulose membrane (Amersham Biosciences) by electroblotting. The membrane was blocked with 5% nonfat dry milk in 1X TBST (50 mM Tris, pH 7.5, 150 mM NaCl, 0.05% Tween-20) for 1 h at room temperature or overnight at 4°C. The membrane was then incubated with primary antibodies for 1 h at room temperature or overnight at 4°C with 5% nonfat dry milk in 1X TBST or 5% bovine serum albumin (BSA) in 1X TBST according to the manufacturer's instructions. After washing three times with 1X TBST for 10 min each, the membrane was incubated with horseradish peroxidase-conjugated secondary antibody (Amersham Biosciences) for 1 h at room temperature. After further washing in 1X TBST three times for 10 min each, the proteins were detected by the enhanced chemiluminescence system (Amersham Biosciences).

### Cell death assays

DNA fragmentation assays were performed on cells treated with either MK-0646 or OSI-906 under GFDS. Cells were seeded in 96-well plates and allowed to grow to 70–80% confluence. The cells were then changed to Supplemented McCoy's medium (‘SM’) and treated with various concentrations of either MK-0646 or OSI-906 for 48 h. Assays were then performed using a cell death ELISA kit (Roche Applied Science) according to the manufacturer's protocol as previously described (21). The plate was read at 405 nm. Inhibition of proliferation was assessed by the MTT [3-(4,5-dimethylthiazol-2-yl)-2,5-diphenyltetrazolium bromide] assay as previously described (22).

### Cell cycle arrest analysis

GEO CRC cells were allowed to grow until 70–80% confluent in 100-mm culture plates and were treated with different concentrations of either MK-0646 or OSI-906 under growth factor deprivation status (GFDS) for 48 h and cell cycle analysis was performed as previously described (23).

### Hematoxylin and eosin, TUNEL and Ki67 staining

Xenografts obtained from subcutaneous injection of GEO cells were harvested and placed in 10% neutral buffer formalin fixative for 12–24 h and then embedded in paraffin. Sections (4 μm) were cut from paraffin-embedded blocks using a microtome and were used for hematoxylin and eosin stains and immunohistochemical characterizations. Serial sections were cut to complement the hematoxylin and eosin sections and were stained with the Apo-tag (Millipore) terminal nucleotidyl transferase mediated nick end-labeling (TUNEL) kit following the manufacturer's protocol. The apoptotic rate was determined semi-quantitatively by counting the number of positively stained apoptotic bodies per 75-μm^2^ field at a magnification of ×20. Approximately 1000 total cells were counted and the percentages of positively stained cells were calculated. Four control and four treated slides for MK-0646 and OSI-906 were analyzed, respectively. Staining was also performed with IgG_1_ rabbit polyclonal antibody for Ki67 (Dako Corp.). Ki67 is a non-histone nuclear antigen present in late G_1_, G_2_, and S phases of the cell cycle but not in G_0_. A 1:20 dilution was used and staining was performed following the manufacturer's protocol. The proliferation rate was determined semi-quantitatively by counting the number of positively stained proliferative cells per 75-μm^2^ field at a magnification of ×20. Approximately 1000 total cells were counted and the percentages of positively stained cells were calculated. Four control and four treated slides for MK-0646 and OSI-906 were analyzed, respectively.

### Immunohistochemistry

Sections (4 μm) were cut from the paraffin-embedded xenograft tumor blocks, deparaffinized in histoclear, and rehydrated in descending grades of ethanol. Endogenous peroxidase activity was blocked with 3% hydrogen peroxide in water. Immunostaining was performed for XIAP using an indirect detection method (24). The staining was accompanied by a negative control in which slides were incubated with a matching blocking peptide to the primary antibody. Slides were counterstained with hematoxylin. Specimens were processed on the same day to eliminate any variability in conditions. Slides were digitally photographed using the same settings.

### Statistical analysis

Statistical significance was determined using two-tailed Student's t-test with a p-value <0.05. All the experiments were repeated three times independently to determine consistency in the results. The results were expressed as mean ± SE for three replicates for each treatment.

## Results

### Inhibition of IGF-1R is associated with antitumor activity in vivo

**Figure 1:**
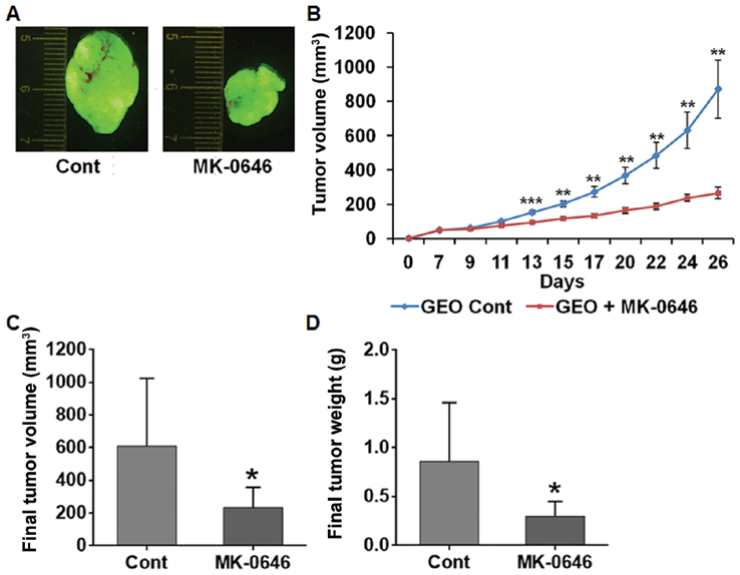
*In vivo* analysis of IGF-1R inhibition using MK-0646 on tumor growth and weight in GEO CRC xenografts. Significant decrease of the tumor volume and weight was observed in MK-0646 treated xenografts compared with the control (A, B, C and D). (n=14, control=7, MK-0646 treated=7).

**Figure 2:**
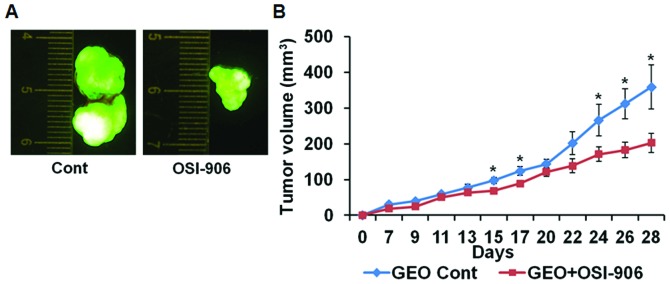
*In vivo* analysis of IGF-1R inhibition using OSI-906 on tumor growth and weight in GEO CRC xenografts. Significant decrease of the tumor volume was observed in OSI-906 treated xenografts compared with the control (A and B). (n=9, control=4, OSI-906 treated=5).

The antitumor effects were determined in the IGF-1R-dependent CRC sub-cutaneous xenograft tumors following treatment with IGF-1R antagonist MK-0646 or OSI-906. MK-0646 treatment for three weeks at 20 mg/kg dose once weekly inhibited the growth of the GEO xenograft tumors (Fig. 1A and B). Similar results were obtained in MK-0646 treated CBS xenograft tumor (data not shown). The final tumor volume and weight were significantly reduced in MK-0646 treated xenografts compared with the control (Fig. 1C and D). Results obtained by daily treatment of OSI-906 (40 mg/kg) for two weeks orally (Fig. 2A and B) were comparable to the MK-0646 treated tumor xenografts showing decrease in tumor growth. However, we observed ~10% body weight reduction in OSI-906 treated mice compared with the MK-0646 treated mice (data not shown), which may be attributed to inhibition of insulin receptor by the dual kinase inhibitor.

### IGF-1R inhibition of apoptosis in vivo and in vitro

**Figure 3:**
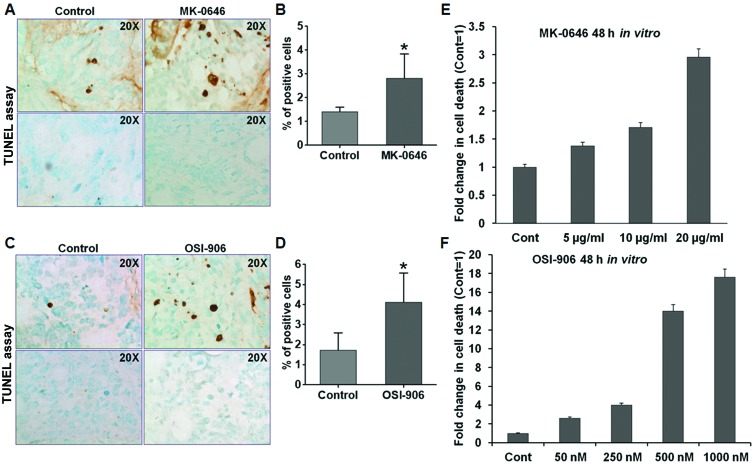
IGF-1R inhibition on apoptosis *in vivo* and *in vitro*. TUNEL assay was performed on both MK-0646 and OSI-906 treated and control xenograft paraffin-embedded slides and statistical analysis was determined (A, B, C and D). GEO cells were grown to 70–80% confluency and treated with different doses of either MK-0646 or OSI-906 under GFDS for 48 h and performed DNA fragmentation assay as explained in Materials and methods (E and F).

We assessed the apoptosis level of control and drug-treated xenografts using TUNEL assays. Comparable results were obtained for both IGF-1R antagonists. Both MK-0646 and OSI-906 treated GEO xenografts had statistically significant increase in apoptosis (p<0.05) as compared with the control tumors (Fig. 3A–D). Based on the pro-apoptotic effects of both MK-0646 and OSI-906 *in vivo*, DNA fragmentation was performed *in vitro* on GEO CRC cells to determine cell death following IGF-1R antagonist treatment (Fig. 3E and F). In accordance with the *in vivo* study, both MK-0646 and OSI-906 treatment showed significant increases in apoptosis demonstrating that IGF-1R inhibition elicits pro-apoptotic effects on CRC cells.

### IGF-1R inhibition of cell proliferation in vivo and in vitro

**Figure 4:**
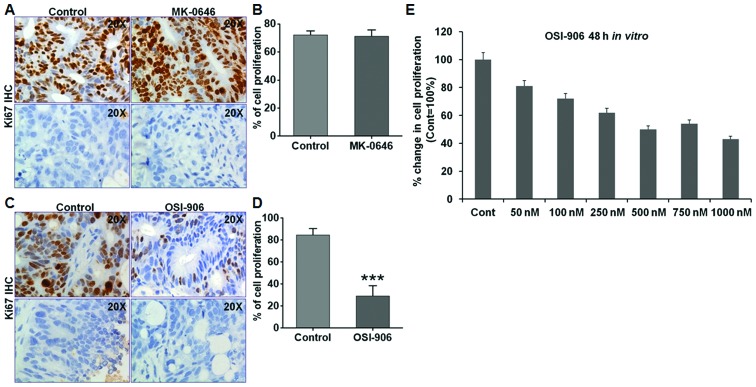
IGF-1R inhibition on cell proliferation *in vivo* and *in vitro*. Ki-67 staining was performed on both MK-0646 and OSI-906 treated and control xenograft paraffin-embedded slides and statistical analysis was determined (A, B, C and D). In order to determine the *in vitro* effects of IGF-1R inhibition on proliferation, MTT assay was performed with different doses of OSI-906 under GFDS for 48 h (E).

**Figure 5:**
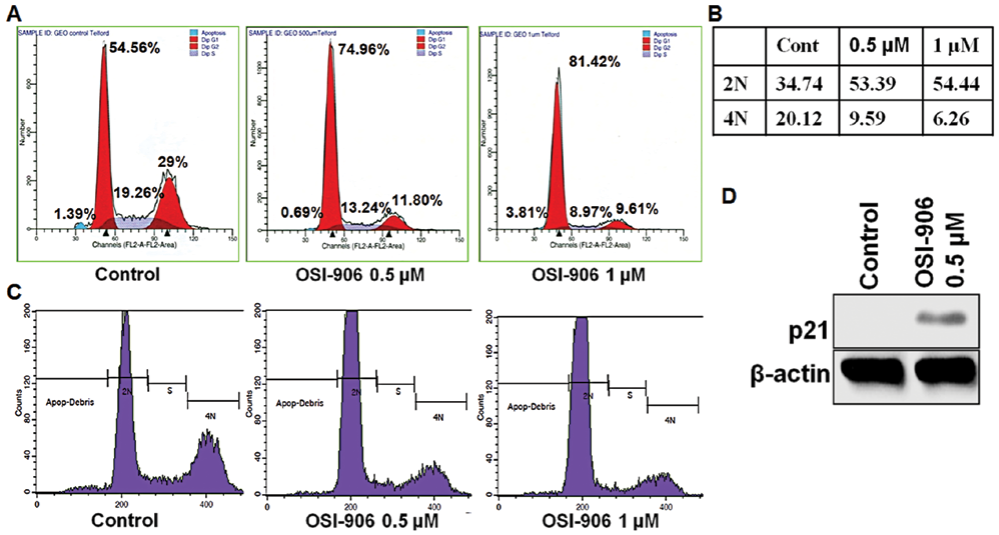
Effects of IGF-1R inhibition on cell cycle *in vitro*. GEO CRC cells were grown to 70–80% confluence treated with OSI-906 under GFDS for 48 h and performed cell cycle analysis as explained in Materials and methods (A–C). Western blot analysis was performed on the OSI-906 treated GEO CRC cell lysates with p21 antibody using actin as a loading control (D).

Ki67 staining was performed to assess the proliferation level on MK-0646 and OSI-906 treated GEO xenografts. MK-0646 treated GEO xenografts showed no change in the cell proliferation compared with the control (Fig. 4A and B). However, OSI-906 treated GEO xenografts showed a statistically significant reduction (p<0.05) in cell proliferation compared with the control (Fig. 4C and D). We also assessed cell proliferation of GEO CRC cells *in vitro* by MTT assay after treating with different concentrations of MK-0646 or OSI-906. OSI-906 treatment showed a decrease in cell proliferation (Fig. 4E), altered cell cycle in G_0_/G_1_ phase (Fig. 5A) and showed a decrease in the 4N DNA content (Fig. 5B and C). However, MK-0646 treatment showed no effect on cell cycle (data not shown). OSI-906 treatment also led to increase in p21 expression (Fig. 5D).

### IGF-1R treatment in vivo and in vitro decreases downstream substrates

**Figure 6:**
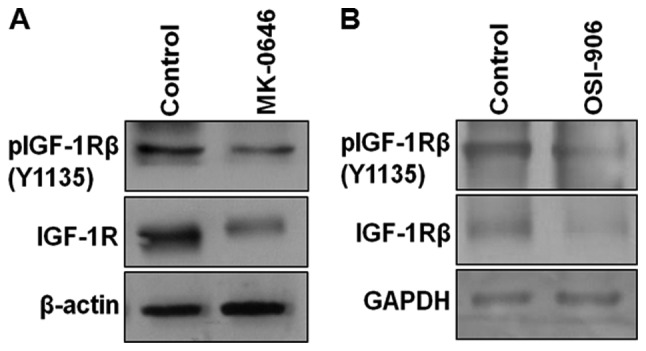
IGF-1R inhibition *in vivo* and *in vitro* decreases downstream substrates. GEO xenografts of both MK-0646 and OSI-906 were homogenized and western blot analyses were performed with antibodies to IGF-1Rβ and pIGF-1Rβ (Y^1135^). Actin and GAPGH were used as loading controls (A and B).

Previous studies have shown that IGF-1R signaling exerts its anti-apoptotic effect through the IRS1/IRS2/PI3K/Akt pathway (25–28). We determined the effects of MK-0646 and OSI-906 on the IGF-1R and its downstream signaling pathways on control and treated GEO CRC xenografts. The xenograft tumor samples were analyzed for the expression of molecules associated with the IGF-1R signaling pathway, including IGF-1Rβ (Y^1135^), Akt and pAkt (S^473^). Inhibition of IGF-1R by MK-0646 and OSI-906 led to the downregulation of IGF-1R and its phosphorylation (Fig. 6A and B), confirming the inhibitory effect of both antagonists on IGF-1R signaling. IGF-1R inhibition by MK-0646 or OSI-906 showed dephosphorylation of Akt at S^473^ site (data not shown). We next determined the effects of IGF-1R inhibition *in vitro*, using GEO cells following treatment with either MK-0646 or OSI-906. GEO cells were treated with MK-0646 or OSI-906 under GFDS conditions. Both MK-0646 and OSI-906 showed similar response on IGF-1R and p-IGF-1R *in vitro* (data not shown). Additionally, marked reduction in pAkt (S^473^) was also observed in MK-0646 (20 μg/ml) or OSI-906 (0.5 μM) treated cells (data not shown). These results showed that both antagonists MK-0646 and OSI-906 effectively inhibited IGF-1R and its downstream signaling.

### IGF-1R inhibition downregulates the IAP molecule XIAP in vivo and in vitro

**Figure 7:**
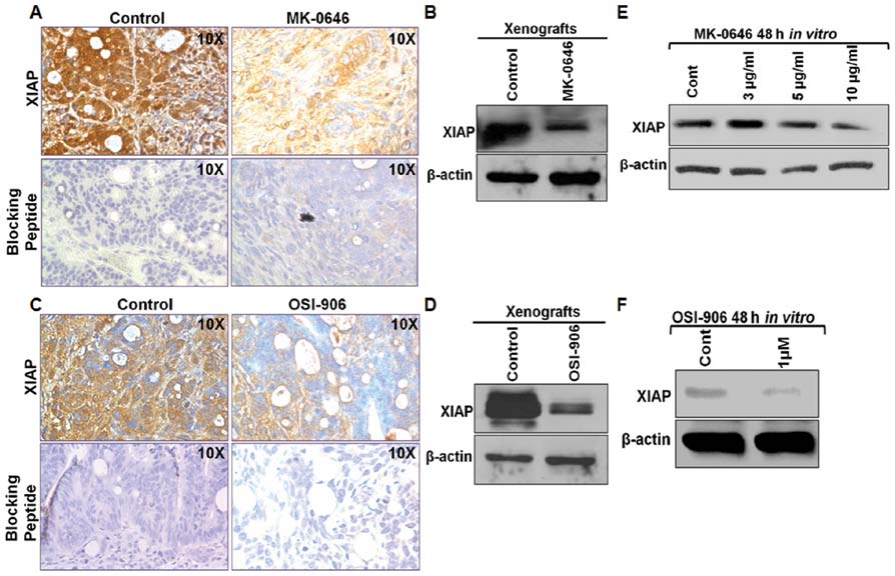
IGF-1R inhibition downregulates the IAP molecule XIAP *in vivo* and *in vitro*. Immunohistochemical analysis of XIAP protein was performed on both MK-0646 and OSI-906 treated paraffin-embedded xenografts. To confirm antibody specificity, a blocking peptide was used (A and B). Control and MK-0646 or OSI-906 treated GEO xenografts were analyzed by western blotting using anti-XIAP antibody (C and D). For *in vitro* analysis, GEO CRC cells were grown to 70–80% confluence and treated with either MK-0646 or OSI-906 under GFDS for 48 h. Cells were harvested as described in Materials and methods and western blot analyses were performed with antibody to XIAP. Actin was used as a loading control (E and F).

XIAP, an IAP (inhibitor of apoptosis) molecule and a key cell survival protein for inhibition of caspases, is a physiological substrate of Akt (29). Akt phosphorylates XIAP at Ser^87^ and regulates its autoubiquitination and degradation, and thereby stabilizes XIAP (29). Chowdhury *et al* have shown that XIAP is associated with pAKT and this association is disrupted following TGFβ treatment leading to XIAP degradation (30). Moreover, XIAP and survivin form a complex in the cytosol and this complex inhibits caspase activity as well as cell death and promotes tumor growth *in vivo*(31). Inhibition of the aberrant cell survival signaling of XIAP through destabilization of XIAP/survivin complexes leads to caspase reactivation and cell death (30,32). We demonstrated that IGF-1R signaling pathway inhibition either by MK-0646 or OSI-906 both *in vivo* and *in vitro* downregulated Akt signaling, and this inhibition in turn leads to the downregulation of XIAP by immunohistochemical and western blot analysis (Fig. 7A–D). Next, we treated GEO cells with the IGF-1R antagonist *in vitro* for 48 h. Similar to the *in vivo* results, MK-0646 and OSI-906 both showed XIAP downregulation in the treated lysates compared to the control (Fig. 7E and F). These results showed that both antagonists exhibited their pro-apoptotic mechanism through inhibition of XIAP, an important downstream cell survival pathway molecule required for the survival of the IGF-1R-dependent CRC cells.

## Discussion

Aberrant regulation of growth factors and their corresponding receptors play important roles in malignant progression (33–38). IGF-1R signaling pathway is prevalent in many cancers, including CRC (39–41). The IGF-IR gene has been reported to be overexpressed in human CRC (36) with ~30–40% of all CRC being IGF-1R-dependent (22,42). Therefore, IGF-1R signaling pathway is under intense investigation as an attractive candidate for the development of novel therapeutic strategies for anticancer treatment.

Buck *et al* inhibited the IGF-1R signaling pathway using OSI-906 in GEO xenograft tumors (18). It was reported that OSI-906 inhibited the growth of the GEO xenografts (18). Recently, we demonstrated that IGF-1R kinase inhibitor PQIP causes marked antitumor activity in these colon cancer cell lines by abrogating the IGF-1R mediated activation of IRS1/Akt to inhibit survival signaling, and inducing apoptosis (10). In the present study, we analyzed the antitumor activity of a novel recombinant humanized monoclonal antibody, MK-0646 in CRC cells both *in vivo* and *in vitro* in comparison to OSI-906. Monoclonal antibodies against IGF-1R share a common mechanism of action, involving blockade of ligand-receptor interactions and decreased cell surface receptor through receptor internationalization and downregulation of the receptor (11,43–45). This leads to blockade of the PI3K/Akt signaling pathway (43–46). However, mechanisms associated with IGF-1R antagonist-mediated cell death are poorly understood. Our data demonstrated that MK-0646 decreased tumor growth in CRC xenografts *in vivo* and is supported by downregulation of IGF-1R and pIGF-1Rβ in western blot analysis. MK-0646 demonstrated similar antitumor activity when compared to OSI-906.

IGF-1R signaling exerts its anti-apoptotic effect through IRS1/IRS2/PI3K/Akt pathway (10,22,25–28). We observed downregulation of IRS-1/2 and pAkt (S^473^) and significant increase in tumor cell apoptosis after MK-0646 or OSI-906 treatment. Akt and its downstream molecular targets constitute a major cell survival pathway (47). XIAP, a pro-survival IAP, is a physiological substrate of Akt (29). Akt phosphorylates XIAP at Ser^87^ and reduces its degradation conferring resistance to caspase activation and apoptosis (29). Deregulation of IAP functions aberrantly prolonging cancer cell viability, and XIAP and survivin have been recognized for their role in tumor formation and are targets for cancer therapeutics (31). We made the novel observation that XIAP, a critical cell survival molecule that counteracts caspase activation and induction of apoptosis (29,31) is downregulated with both MK-0646 and OSI-906 treatments demonstrating that XIAP is downstream of IGF-1R/Akt mediated control of aberrant cell survival responses. XIAP has been linked to cell survival and metastasis (48). XIAP/survivin complexes that mediate caspase inhibition have been shown to be a key cell survival mechanism for supporting the metastatic process (31). Previous studies in our laboratory have shown that destabilization of XIAP/survivin complexes by the TGFβ tumor suppressor signaling leads to inhibition of aberrant cell survival resulting in cell death (30,32). Therefore, the IGF-1R signaling pathway and its downstream cell survival mediator XIAP may be potential dual targets for anticancer therapy.

In conclusion, this study demonstrated that MK-0646, a novel humanized IGF-1R monoclonal antibody, has comparable antitumor effects to IGF-1R small molecule inhibitor OSI-906, and may be a potential novel targeted therapy against IGF-1R-dependent subset of human CRC. Therefore, the results obtained in this study utilizing IGF-1R antagonists provide a rationale for further pre-clinical studies in order to dissect the IGF-1R signaling pathway to obtain full benefit from this receptor targeted therapy as a single agent or in combination against CRC as well as other solid tumors dependent upon IGF-1R signaling.
